# Author Correction: Investigation of Cas9 antibodies in the human eye

**DOI:** 10.1038/s41467-022-29844-x

**Published:** 2022-04-12

**Authors:** Marcus A. Toral, Carsten T. Charlesworth, Benjamin Ng, Teja Chemudupati, Shota Homma, Hiromitsu Nakauchi, Alexander G. Bassuk, Matthew H. Porteus, Vinit B. Mahajan

**Affiliations:** 1 Molecular Surgery Program, Department of Ophthalmology, Byers Eye Institute, Stanford University, Palo Alto, CA USA; 2 Medical Scientist Training Program and Graduate Program in Molecular Medicine, University of Iowa, Iowa City, IA USA; 3 Department of Pediatrics, Stanford University, Palo Alto, CA USA; 4 Medical Sciences Division, University of Oxford, Oxford, UK; 5 Department of Genetics, Stanford University, Palo Alto, CA USA; 6 Institute for Stem Cell Biology and Regenerative Medicine, Stanford University School of Medicine, Palo Alto, CA USA; 7 Division of Stem Cell Therapy, Distinguished Professor Unit, Institute of Medical Science, University of Tokyo, Tokyo, Japan; 8 Departments of Pediatrics and Neurology and The Iowa Neuroscience Institute (INI), University of Iowa, Iowa City, IA USA; 9 Veterans Affairs Palo Alto Health Care System, Palo Alto, CA, Palo Alto, CA USA

**Keywords:** CRISPR-Cas systems, Visual system, Translational immunology

Correction to: *Nature Communications* 10.1038/s41467-022-28674-1, published online 25 February 2022.

The original version of this article contained errors in the Methods section, where the incorrect suppliers for two antibodies were listed. The sentence ‘HRP-conjugated goat anti-human Fc antibody (Novus)’ should read ‘HRP-conjugated goat anti-human Fc antibody (Bethyl Laboratories)’, and the sentence ‘HRP-conjugated goat anti-mouse Fc antibody (Novus)’ should read ‘HRP-conjugated goat anti-mouse Fc antibody (GE Healthcare)’. These errors have now been corrected in both the PDF and HTML versions of the Article.

