# Supplementary material for: Investigation of Cas9 antibodies in the human eye
Source: Nat Commun. 2022 Feb 25;13:1053. doi: 10.1038/s41467-022-28674-1 (PMC8881612; doi:10.1038/s41467-022-28674-1)
Supplement: Supplementary file 1 — Supplementary Information [file 41467_2022_28674_MOESM1_ESM.pdf]

Supplemental Information

Investigation of Cas9 Antibodies in the Human Eye

Toral *et al.*

This document contains:

1. Supplemental Table 1: Human subject cohort information
2. Supplemental Table 2: Antibody information

Supplementary Table 1. Human subject cohort information.

**Cohort 1 (*n* = 13) Summary Details**			
Sex Ratio (M:F)	10:3		
Mean Age (s.d.)	M: 64.7 years (14.4)<br>F: 68.7 years (14.4)		
Median Age	M: 70 years<br>F: 77 years		
**Case**	**Age (years)**	**Ocular Diagnosis**	**Cohort**
#1	74	Macular hole, posterior intraocular lens	Paired Vitreous & Serum (cohort 1)
#2	77	Vitreous floater	Paired Vitreous & Serum (cohort 1)
#3	26	Congenital cataracts, posterior intraocular lens	Paired Vitreous & Serum (cohort 1)
#4	77	Intraretinal hemorrhage, epiretinal membrane	Paired Vitreous & Serum (cohort 1)
#5	71	Epiretinal membrane, posterior vitreous detachment, retinal edema	Paired Vitreous & Serum (cohort 1)
#6	73	Epiretinal membrane, posterior vitreous detachment	Paired Vitreous & Serum (cohort 1)
#7	69	Epiretinal membrane, anterior hyalosis	Paired Vitreous & Serum (cohort 1)
#8	66	Choroidal melanoma, retinal detachment, posterior vitreous detachment, vitreous hemorrhage	Paired Vitreous & Serum (cohort 1)
#9	62	Proliferative diabetic retinopathy, vitreous hemorrhage	Paired Vitreous & Serum (cohort 1)
#10	52	Retinal detachment, vitreous hemorrhage	Paired Vitreous & Serum (cohort 1)
#11	61	Cone-rod dystrophy, epiretinal membrane	Paired Vitreous & Serum (cohort 1)
#12	74	Retinitis pigmentosa, epiretinal membrane	Paired Vitreous & Serum (cohort 1)
#13	71	Posterior vitreous detachment, vitreous hemorrhage	Paired Vitreous & Serum (cohort 1)
**Cohort 2 (*n* = 36) Summary Details**			
Sex Ratio (M:F)	5:4<br>(total: 25 male, 16 female)		
Mean Age (s.d.)	M: 63.4 years (13.3)<br>F: 64.0 years (12.2)		
Median Age	M: 62.5 years<br>F: 66 years		
**Case**	**Age (years)**	**Ocular Diagnosis**	**Cohort**

#14	70	Epiretinal membrane	Unpaired Vitreous (cohort 2)
#15	34	Proliferative diabetic retinopathy, vitreous hemorrhage, retinal detachment	Unpaired Vitreous (cohort 2)
#16	62	Vitreous floater	Unpaired Vitreous (cohort 2)
#17	67	Macular hole	Unpaired Vitreous (cohort 2)
#18	81	Epiretinal membrane, macular hole	Unpaired Vitreous (cohort 2)
#19	52	Vitreous hemorrhage, epiretinal membrane, cystoid macular edema	Unpaired Vitreous (cohort 2)
#20	70	Posterior vitreous detachment	Unpaired Vitreous (cohort 2)
#21	61	Macular hole	Unpaired Vitreous (cohort 2)
#22	86	Retinal detachment	Unpaired Vitreous (cohort 2)
#23	48	Proliferative diabetic retinopathy	Unpaired Vitreous (cohort 2)
#24	67	Macular hole, retinal detachment	Unpaired Vitreous (cohort 2)
#25	48	Proliferative diabetic retinopathy	Unpaired Vitreous (cohort 2)
#26	63	Epiretinal membrane, posterior vitreous detachment	Unpaired Vitreous (cohort 2)
#27	68	Retinal detachment	Unpaired Vitreous (cohort 2)
#28	57	Posterior vitreous detachment, cystoid macular edema	Unpaired Vitreous (cohort 2)
#29	66	Epiretinal membrane	Unpaired Vitreous (cohort 2)
#30	75	Epiretinal membrane, cystoid macular edema	Unpaired Vitreous (cohort 2)
#31	72	Recurrent macular hole, epiretinal membrane	Unpaired Vitreous (cohort 2)
#32	58	Epiretinal membrane	Unpaired Vitreous (cohort 2)
#33	56	Epiretinal membrane, macular hole	Unpaired Vitreous (cohort 2)
#34	66	Epiretinal membrane, macular hole, posterior vitreous detachment	Unpaired Vitreous (cohort 2)
#35	58	Proliferative diabetic retinopathy, retinal edema	Unpaired Vitreous (cohort 2)
#36	84	Retinal detachment	Unpaired Vitreous (cohort 2)
#37	58	Epiretinal membrane, posterior vitreous detachment	Unpaired Vitreous (cohort 2)
#38	72	Macular hole	Unpaired Vitreous (cohort 2)

#39	85/F	Macular hole, posterior vitreous detachment	Unpaired Vitreous (cohort 2)
#40	32/M	Proliferative diabetic retinopathy, vitreous hemorrhage	Unpaired Vitreous (cohort 2)
#41	62/M	Proliferative diabetic retinopathy, vitreous hemorrhage	Unpaired Vitreous (cohort 2)
#42	65/F	Vitreous hemorrhage, retinal tear	Unpaired Vitreous (cohort 2)
#43	56/F	Retinal detachment, epiretinal membrane	Unpaired Vitreous (cohort 2)
#44	69/M	Retinal detachment	Unpaired Vitreous (cohort 2)
#45	84/M	Endophthalmitis, epiretinal membrane, age-related macular degeneration	Unpaired Vitreous (cohort 2)
#46	48/F	Proliferative diabetic retinopathy, vitreous hemorrhage	Unpaired Vitreous (cohort 2)
#47	59/M	Retinal detachment	Unpaired Vitreous (cohort 2)
#48	70/F	Epiretinal membrane, retinal edema	Unpaired Vitreous (cohort 2)
#49	62/F	Retinal detachment, cataract	Unpaired Vitreous (cohort 2)

Supplemental Table 2. Antibody information.

**Antibody**	**Manufacturer**	**Product number**	**Dilution Used**
Goat anti-Human IgG Fc Secondary Antibody	Bethyl Laboratories	A80-104	1:100,000
Sheep anti-Mouse IgG Fc Secondary Antibody	GE Healthcare	NA931V	1:10,000
