# Supplementary material for: Investigation of Cas9 antibodies in the human eye
Source: Nat Commun. 2022 Feb 25;13:1053. doi: 10.1038/s41467-022-28674-1 (PMC8881612; doi:10.1038/s41467-022-28674-1)
Supplement: Supplementary file 2 — Reporting summary [file 41467_2022_28674_MOESM2_ESM.pdf]

nature portfolio

Corresponding author(s): Vinit B. Mahajan

Last updated by author(s): Jan 13, 2022

Reporting Summary

Nature Portfolio wishes to improve the reproducibility of the work that we publish. This form provides structure for consistency and transparency in reporting. For further information on Nature Portfolio policies, see our Editorial Policies and the Editorial Policy Checklist.

Statistics

For all statistical analyses, confirm that the following items are present in the figure legend, table legend, main text, or Methods section.

n/a	Confirmed	
☐	☒	The exact sample size (*n*) for each experimental group/condition, given as a discrete number and unit of measurement
☐	☒	A statement on whether measurements were taken from distinct samples or whether the same sample was measured repeatedly
☐	☒	The statistical test(s) used AND whether they are one- or two-sided<br>*Only common tests should be described solely by name; describe more complex techniques in the Methods section.*
☐	☒	A description of all covariates tested
☐	☒	A description of any assumptions or corrections, such as tests of normality and adjustment for multiple comparisons
☐	☒	A full description of the statistical parameters including central tendency (e.g. means) or other basic estimates (e.g. regression coefficient) AND variation (e.g. standard deviation) or associated estimates of uncertainty (e.g. confidence intervals)
☐	☒	For null hypothesis testing, the test statistic (e.g. *F*, *t*, *r*) with confidence intervals, effect sizes, degrees of freedom and *P* value noted<br>*Give P values as exact values whenever suitable.*
☒	☐	For Bayesian analysis, information on the choice of priors and Markov chain Monte Carlo settings
☒	☐	For hierarchical and complex designs, identification of the appropriate level for tests and full reporting of outcomes
☒	☐	Estimates of effect sizes (e.g. Cohen's *d*, Pearson's *r*), indicating how they were calculated

Our web collection on statistics for biologists contains articles on many of the points above.

Software and code

Policy information about availability of computer code

Data collection	No software was used for data collection.
Data analysis	PRISM 9.3.1 (GraphPad)<br>MasterPlex (Hitachi Software Engineering America Ltd., MiraiBio Group)

For manuscripts utilizing custom algorithms or software that are central to the research but not yet described in published literature, software must be made available to editors and reviewers. We strongly encourage code deposition in a community repository (e.g. GitHub). See the Nature Portfolio guidelines for submitting code & software for further information.

Data

Policy information about availability of data

All manuscripts must include a data availability statement. This statement should provide the following information, where applicable:
- Accession codes, unique identifiers, or web links for publicly available datasets
- A description of any restrictions on data availability
- For clinical datasets or third party data, please ensure that the statement adheres to our policy

All data analyzed or generated during this study are available within the article in the Source Data or Supplemental Information files.

Field-specific reporting

Please select the one below that is the best fit for your research. If you are not sure, read the appropriate sections before making your selection.

☒ Life sciences ☐ Behavioural & social sciences ☐ Ecological, evolutionary & environmental sciences

For a reference copy of the document with all sections, see nature.com/documents/nr-reporting-summary-flat.pdf

Life sciences study design

All studies must disclose on these points even when the disclosure is negative.

Sample size

For human studies of human biopsies, the maximum amount of samples were included which could be obtained. For mouse studies, a sample size of n = 10 mice per experiment was used, consistent with the mouse literature and anticipated effect sizes. For our typical mouse studies, a sample size of 8 mice yields 75% power to detect at least a modest effect, whereas a sample of 10 yields 85% power to detect the same. In studies of wild-type C57BL/6J mice, which typically display low levels of phenotypic variability, sample sizes of at least n = 5 are common in the literature. Thus, we chose sample sizes of n = 10 to optimize scientific rigor and using the fewest mice necessary.

Wert KJ, Skeie JM, Bassuk AG, Olivier AK, Tsang SH, Mahajan VB. Functional validation of a human CAPN5 exome variant by lentiviral transduction into mouse retina. Hum Mol Genet. 2014;23(10):2665-77. doi: 10.1093/hmg/ddt661. PubMed PMID: 24381307.

Wert KJ, Bassuk AG, Wu WH, Gakhar L, Coglan D, Mahajan M, Wu S, Yang J, Lin CS, Tsang SH, Mahajan VB. CAPN5 mutation in hereditary uveitis: the R243L mutation increases calpain catalytic activity and triggers intraocular inflammation in a mouse model. Hum Mol Genet. 2015;24(16):4584-98. doi: 10.1093/hmg/ddv189. PubMed PMID: 25994508.

Dell RB, Holleran S, Ramakrishnan R. Sample size determination. ILAR J. 2002;43(4):207-13. PubMed PMID: 12391396.

Data exclusions

No data were excluded from the analyses.

Replication

For human immunoglobulin isotyping, replicates of 2 were performed on all samples. For all ELISA experiments using human serum or vitreous fluid, or mouse serum replicates of 2-3 were performed. For ELISA experiments using mouse vitreous fluid, no replication was possible due to limitations on sample volume. All attempts at replication were successful.

Randomization

Human and mouse samples were coded and randomized.

Blinding

Samples were coded and investigators were blinded to samples during data collection and analysis for all experiments.

Reporting for specific materials, systems and methods

We require information from authors about some types of materials, experimental systems and methods used in many studies. Here, indicate whether each material, system or method listed is relevant to your study. If you are not sure if a list item applies to your research, read the appropriate section before selecting a response.

Materials & experimental systems

n/a	Involved in the study	
☐	☒	Antibodies
☒	☐	Eukaryotic cell lines
☒	☐	Palaeontology and archaeology
☐	☒	Animals and other organisms
☐	☒	Human research participants
☒	☐	Clinical data
☒	☐	Dual use research of concern

Methods

n/a	Involved in the study	
☒	☐	ChIP-seq
☒	☐	Flow cytometry
☒	☐	MRI-based neuroimaging

Antibodies

Antibodies used

Goat anti-Human IgG Fc Secondary Antibody, HRP-conjugated (Novus Biologicals; NB7449), used at 1:100,000 dilution
Goat anti-Mouse IgG Fc Secondary Antibody, HRP-conjugated (Novus Biologicals; NB7561), used at 1:10,000 dilution

Validation

No primary antibodies were used in the study.

Both Novus Biologicals antibodies used were validated in-house and by Novus Biologicals for use in ELISAs. Additionally, both antibodies have been published on in multiple studies:

Goat anti-Human IgG Fc Secondary Antibody:

- Huang H, Fang L, Xue L et al. PEGylation but Not Fc-Fusion Improves in Vivo Residence Time of a Thermostable Mutant of Bacterial Cocaine Esterase Bioconjug. Chem. Nov 25 2019 [PMID: 31661952]
- Friberg H, Jaiswal S, West K et al. Analysis of human monoclonal antibodies generated by dengue virus-specific memory B cells. Viral Immunol 2012 Oct [PMID: 22934599]
- Khan MN, Sharma SK, Filkins LM et al. PcpA of Streptococcus pneumoniae mediates adherence to nasopharyngeal and lung epithelial cells and elicits functional antibodies in humans. Microbes Infect 2012 Oct [PMID: 22796387]

Goat anti-Mouse IgG Fc Secondary Antibody:
- Uddowla S, Hollister J, Pacheco JM et al. A safe foot-and-mouth disease vaccine platform with two negative markers for differentiating infected from vaccinated animals. J Virol 2012 Nov [PMID: 22915802]
- Munkhjargal T, Aboulaila M, Ueno A et al. Cloning and characterization of histone deacetylase from Babesia bovis. Vet Parasitol 2012 Dec [PMID: 22818786]
- Lin SC, Lin YF, Chong P et al. Broader neutralizing antibodies against H5N1 viruses using prime-boost immunization of hyperglycosylated hemagglutinin DNA and virus-like particles. PLoS One 2012 [PMID: 22720032]

Animals and other organisms

Policy information about studies involving animals; ARRIVE guidelines recommended for reporting animal research

Laboratory animals	8-week old male C57BL6J mice were used. Mice were housed in Stanford University institutional animal care and use committee (IACUC)-approved facilities, providing 12hr/12hr light/dark cycles, temperatures of 18-23 degrees Celsius, and 40-60% humidity. Food and water were made available at all times and handling, noise and vibrations, and odors were kept to a minimum. A veterinarian was on call 24 hours per day and animals were observed daily by vivarium staff to ensure adequate health and welfare. All mouse experiments described were conducted in accordance with approved Stanford University IACUC protocols and the ARVO Statement for the Use of Animals in Ophthalmic and Vision Research.
Wild animals	No wild animals were used in this study.
Field-collected samples	No field-collected samples were used in this study.
Ethics oversight	All mouse experiments described were conducted in accordance with approved Stanford University IACUC protocols and the ARVO Statement for the Use of Animals in Ophthalmic and Vision Research.

Note that full information on the approval of the study protocol must also be provided in the manuscript.

Human research participants

Policy information about studies involving human research participants

Population characteristics	The study population consisted of two human cohorts of patients undergoing vitreoretinal surgery for a variety of eye diseases (for a full description, see Supplemental Table 1). Cohort 1 was comprised of thirteen subjects (M:F = 10:3) with a mean age of 64.7 years for male participants and 68.7 years for female participants. Cohort 2 was comprised of thirty-six subjects (M:F = 5:4) with a mean age of 63.4 years for male participants and 64.0 years for female participants.
Recruitment	Patients who were undergoing vitreoretinal surgery between September 2019 and March 2020 were recruited by clinical research staff in the ophthalmology clinic. Patients were recruited by explaining the nature and purpose of the study, followed by obtaining written and informed consent from all participants. Given this recruitment approach, there will be self-selection bias in our study population toward individuals able to access surgical ophthalmic care with the education, time available, and communication abilities necessary to fully understand the study. We expect that this may bias the study toward different antibody levels than seen in the general population overall; particularly antibodies against tetanus toxoid, as these are the product of immunization managed through medical providers. Additionally, we expect that the study is also biased toward older individuals and toward men, as patients in our study had a mean age of over 60 years and more male patients than female. Older individuals may exhibit waning immunity, or increased number of lifetime exposure to relevant microbes, leading to lower or higher antibody levels than might be seen in the general population more broadly. We do not expect there to be differences in antibody prevalence between men and women for antibodies tested for in this study, although these differences remain possible. All attempts were made to include a diverse study population representative of the general population given the restrictions of study recruitment.
Ethics oversight	This study obtained Stanford University Institutional Review Board approval and adhered to the tenets set forth in the Declaration of Helsinki.

Note that full information on the approval of the study protocol must also be provided in the manuscript.
